# A New, Carbon-Negative Precipitated Calcium Carbonate Admixture (PCC-A) for Low Carbon Portland Cements

**DOI:** 10.3390/ma12040554

**Published:** 2019-02-13

**Authors:** Lewis McDonald, Fredrik P. Glasser, Mohammed S. Imbabi

**Affiliations:** 1 School of Engineering, University of Aberdeen, Aberdeen AB24 3UE, UK; l.mcdonald.12@aberdeen.ac.uk; 2 Carbon Capture Machine (UK) Limited, Aberdeen AB10 1YL, UK; f.p.glasser@abdn.ac.uk

**Keywords:** CO_2_ emissions, carbon CAPture and CONversion (CAPCON), Precipitated Calcium Carbonate (PCC), limestone, strength, rheology, XRD analysis, Life Cycle Assessment

## Abstract

The production of Portland cement accounts for approximately 7% of global anthropogenic CO_2_ emissions. Carbon CAPture and CONversion (CAPCON) technology under development by the authors allows for new methods to be developed to offset these emissions. Carbon-negative Precipitated Calcium Carbonate (PCC), produced from CO_2_ emissions, can be used as a means of offsetting the carbon footprint of cement production while potentially providing benefits to cement hydration, workability, durability and strength. In this paper, we present preliminary test results obtained for the mechanical and chemical properties of a new class of PCC blended Portland cements. These initial findings have shown that these cements behave differently from commonly used Portland cement and Portland limestone cement, which have been well documented to improve workability and the rate of hydration. The strength of blended Portland cements incorporating carbon-negative PCC Admixture (PCC-A) has been found to exceed that of the reference baseline—Ordinary Portland Cement (OPC). The reduction of the cement clinker factor, when using carbon-negative PCC-A, and the observed increase in compressive strength and the associated reduction in member size can reduce the carbon footprint of blended Portland cements by more than 25%.

## 1. Introduction

### 1.1. Background

Estimates vary, but the cement industry currently produces at least 2.8 billion tonnes of cement per annum, accounting for ~7% of global anthropogenic CO_2_ emissions [1,2,3,4]. Cement production is expected to increase yearly to more than 4 billion tonnes per annum by 2040.

In 2009, the International Energy Agency (IEA) proposed the use of carbon capture and storage to reduce these emissions [5]. Since the IEA’s proposal to reduce the carbon emissions of the cement industry, new technologies have been developed to either capture and utilise post-combustion CO_2_ or convert it into useful products. Some of these technologies are discussed in Section 1.2.

Calcium carbonate, in the form of limestone, has seen an increase in its use as a cement additive to reduce the carbon footprint of cement clinker, which is due to the relatively low CO_2_ output of the process in comparison to Portland cement manufacture [6]. Most codes of practice allow a maximum of 5% ground limestone, containing >70% calcium carbonate, to be added to create Portland limestone cement (PLC) blends as distinct from Ordinary Portland Cement (OPC). Ground limestone has also been demonstrated to increase workability without impacting strength if used in quantities of less than or equal to the maximum allowable 5% in accredited PLC blends [7,8,9].

**Figure 1:**
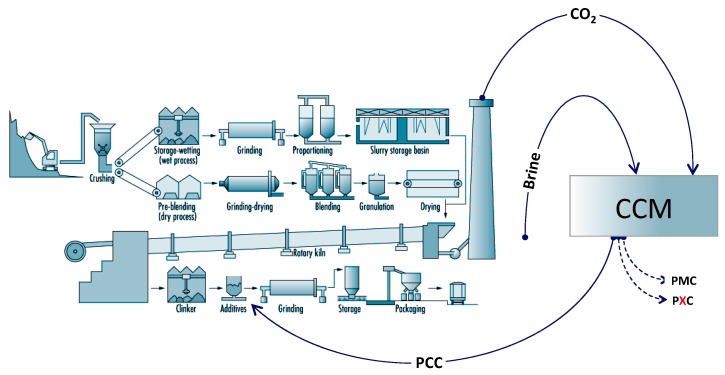
Proposal for the production of a new generation of enhanced performance, low carbon Precipitated Calcium Carbonate Admixture (PCC-A) blended Portland cements can be achieved in situ using the Carbon Capture Machine (CCM).

We present the first results of tests on the use of carbon-negative Precipitated Calcium Carbonate Admixture (PCC-A), a carbon CAPture and CONversion (CAPCON) product of the Carbon Capture Machine (CCM) (see http://www.ccmuk.com for more information), to reduce the carbon footprint of blended cements. Unlike ground limestone, which is carbon positive, PCC-A from the CCM CAPCON process is carbon negative and sequesters between 100 and 350 kgCO_2_ per tonne of PCC-A produced, depending on the process inputs. Our proposal for a more sustainable future, where cement production is decarbonised at source, is illustrated in Figure 1.

The role of calcium carbonate in blended Portland cements spans chemical and physical properties. The chemical role is through the formation of (hemi- and mono-) carboaluminate phases during hydration [10]. Physically, it is predominantly an inert filler [8,11,12,13]. Unpublished data suggests that the control of grain size and the morphology of the PCC-A in the CCM’s CAPCON process can be beneficially used to produce blended Portland cements that do not compromise strength, durability or passivation while offering performance benefits that match or surpass OPC.

The test methods reported in this paper were used to evaluate, for the first time, the effects of adding carbon-negative PCC-A to OPC, potentially leading to the development of new classes of high performance, low carbon Portland cement blends. The methods were used to determine strength, rheology, passivation potential and the reduction in embodied CO_2_ content. It has been shown that the use of PCC-A in blended cements can achieve an increased strength at contents well beyond the 5% limit for ground limestone, while concurrently reducing the embodied CO_2_ content by 25% or more.

### 1.2. Carbon Capture and Utilisation in Cement Production

Portland cement is the most abundantly manufactured material globally and is also one of the largest sources of CO_2_ emissions [1,2,3,4]; it is thus a prime candidate for the emerging carbon capture and utilisation industry to prioritise. A recent report published by Chatham House [14] estimates that, to effectively control the impact of the cement industry on the environment, the clinker factor (i.e., active fraction) in cements has to be reduced to 60% of the present value by 2050. Once this has been achieved, other methods have to be developed that rely on carbon capture and utilisation.

**Figure 2:**
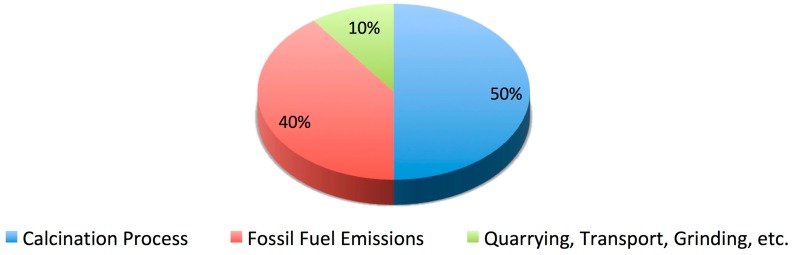
Breakdown of CO_2_ emissions of Portland cement production. Data taken from the Chatham House report [14].

An alternative to carbon capture outlined in the Chatham House report is the use of “novel cements”—low carbon alternatives to traditional Portland cement blends. These cements include allegedly carbon-free magnesium oxide derived from magnesium silicates (MOMS) cements, calcium sulfoaluminate (CSA) cements and carbonated calcium silicate (CCSC) cements amongst others. However, novel cements have seen little market penetration due to their high production costs (MOMS cements), specialty usage (CSA cements), scalability issues (CCSC cements) and resistance to change by codes of practice, consumer groups and standards institutes, compared to tried, tested and trusted Portland cement alternatives. The Chatham House report claims that carbon capture methods could reduce CO_2_ emissions by 95–100%. Similarly, a study of several active carbon capture plants conducted by David and Herzog [15] found the typical efficiency to be 90% for direct emissions. In both cases, the conclusions relate to high cost, non-viable carbon capture and storage routes and are therefore of limited value. The estimates also ignore the contributions of secondary emissions from quarrying, transport and grinding, where the CO_2_ cannot be easily captured—see Figure 2.

Worldwide, CO_2_ capture and utilisation methods for the cement industry currently include the production of carbon-negative admixtures through mineralisation (Carbon Capture Machine) [6] and carbonation cured cements (Solidia, CarbonCure, CO_2_NCRETE) [16,17,18].

Carbonation cured cements were first proposed in the 1970s, but their development has been stunted due to process complexity, cost and application scope. In the carbonation curing process, CO_2_ is converted into the various polymorphs of calcium carbonate—calcite being the most common—through the interaction of CSH gel with CO_2_ (gas) proceeding in two stages: initially the CSH “sheds” Ca forming CaCO_3_ and a low Ca CSH limit is reached at Ca/Si ~1 which, upon continued carbonation, yields silica gel and calcium carbonate [14]. This differs from the normal hydration route where calcium hydroxide is formed in place of calcium carbonate [19]. Prior to CO_2_ exposure, the free water content of the cement is reduced to ensure that the CO_2_ can diffuse efficiently throughout. The cement is then exposed to CO_2_ at pressures up to 5 atm. After exposure, the unreacted cement may be hydrated again to ensure that unreacted phases can continue to hydrate and provide further increases in strength [20]. The CO_2_ content of carbonation cured cements was verified by Seo et al. [21] through XRD analysis and determined to range from 6.9 to 11% of the cement, depending on composition.

The carbonation curing process is claimed to provide cement with enhanced durability, as the calcium carbonate precipitation within the CSH gel creates a denser, less porous structure [22]. However, carbonation reduces the alkalinity of the cement paste and in turn can lead to the early corrosion of reinforcing steel, especially in the presence of chloride ions [23]. The combined reliance on high pressure CO_2_ infusion and the accompanying acidification of the cement binder with consequent passivation loss limits the application scope of such carbonation methods to pre-cast, unreinforced concrete products, such as bricks, blocks and paving tiles, which together account for no more than 20% of all concrete uses [24]. Such cements, however, could potentially be used with non-steel reinforcement, such as glass fibres.

Unlike carbonation curing, mineralisation through carbon capture serves more than one role; the CO_2_ is converted into minerals, such as calcium carbonate and magnesium carbonate, which are used in a multitude of industries. The method of mineralisation can include the carbonation of metal oxide by-products from mining processes [25] or through precipitating the minerals using CO_2_ absorption in an aqueous alkali solution [26]. In the cement industry, calcium carbonate can be used as a clinker replacement, as described in Section 1.1. Based on theory and experience, the maximum efficiency of CO_2_ mineralisation has been estimated at 68% [27].

The use of mineralised CO_2_ in the form of PCC-A (produced through carbon CAPCON technology developed by CCM) as a cement admixture is the focus of this paper, where first results have shown that a significant reduction of CO_2_ emissions of around 25% can be achieved by the substitution of clinker with carbon-negative PCC-A (see Section 3.4). The PCC-A in cement reacts with water during formation to produce hydrated calcium carboaluminates that develop from the hydration and partial carbonation of C_3_A phases. In doing so, the interaction between water and C_3_A is modified, leading to a more rapid formation of ettringite. By stabilising carboaluminates, sulfate is forced to remain in the ettringite and so conversion back to monosulfoaluminate becomes thermodynamically unfavourable [8]. This potentially impacts the role of gypsum, a widely used setting retarder [28]. 

The reference CO_2_ content of an off-the-shelf PLC was determined in the course of the tests, using XRD. At 11.4%, it has a comparable CO_2_ content to carbonation cured cements at 11.0% [21], but with none of the inherent disadvantages or limitations. Further testing of PCC-A blended cements is expected to confirm that a significantly higher embodied CO_2_ content can be achieved without either detriment to quality or limitation to the range of products made and applications deployed, when using these new generation Portland cements.

## 2. Materials and Methods

### 2.1. Materials

Two types of cement were used in the tests carried out—a CEM I 52.5N OPC and a CEM II/A-LL 32.5R PLC. Hanson Cement UK supplied both products. The main constituents of the cements were determined through XRD analysis and Rietveld Refinement, as detailed in Section 2.2 and Section 3.1. The manufacturer specifies that both cements have a Blaine fineness between 300 and 500 m^2^/kg.

The PCC-A used is a product of the carbon CAPCON process developed by Carbon Capture Machine (UK) Limited. It was prepared from CO_2_-rich flue gas, where CO_2_ was selectively dissolved at the point of emission in dilute aqueous alkali (NaOH) solution. The solution was then mixed with calcium-containing brine to produce the PCC-A, which was subsequently filtered, rinsed and dried before use to remove contaminants, such as NaCl.

### 2.2. X-ray Diffraction (XRD) Analysis

Samples of the PCC-A, and both cement types, were tested using a PANalytical X’Pert X-ray powder diffractometer to determine the composition. Powder specimens were analysed at room temperature at a 2θ range from 30° to 80°, Cu K alpha radiation, using a scan period of 10 min.

### 2.3. Rheological Data

The rheological data of the freshly mixed cement pastes were measured using a continually increasing shear rate up to 200 1/s over a period of 100 s before decreasing at the same rate to 0. A ⌀25 mm coaxial cylinder spindle was used in conjunction with a ⌀40 mm sample pot.

The cement and PCC-A were weighed to provide a total solids content of 200 g and the desired water/solids ratio was also weighed. The PCC-A was added to the water and stirred continuously to ensure it was evenly dispersed. The PCC-A/water slurry was then combined with the cement in a mixing bowl and mixed continuously for 90 s; this mixing period was considered sufficient to ensure the hydration of the cement paste while reducing the risk of early-set formation. Within 30 s of the mixing ending, the paste was transferred to the sample pot and connected to the rheometer and the test was started. 

Data were collected for samples of OPC, PLC and PCC-A blended cement ranging from 0 to 20% PCC-A content. As the water/cement ratio decreased, the range of PCC-A cements that were sufficiently workable to test also decreased.

### 2.4. Compressive Strength

Cement pastes were prepared in the same way as for rheological testing, but with a longer mix time in accordance with BS EN 196-1:2016 [29]. A longer mix time, compared with the rheological tests, was used to ensure the PCC-A was well distributed in the paste before casting. The paste was then cast in 50 × 50 × 50 mm cube steel moulds. Samples were maintained at a constant temperature of 25 °C for 24 h before being demoulded. Samples were thereafter fully submerged in a water bath at an ambient temperature for 7 or 28 days. It was observed during casting that cement pastes containing a high PCC-A content were less workable, i.e., they were more viscous than OPC and at the highest PCC-A content were difficult to cast.

After curing, the samples were wiped dry before being tested. Testing was carried out using a hydraulic ram at a loading rate of 1 mm/min. The applied load was increased until the sample fractured. Data were collected electronically with a measurement of displacement and force recorded 6 times per second. The compressive strength was calculated as a variant of the equation provided in BS EN 196-1:2016 [29] to account for the different cube size tested:

(1) $$R_{c}=\frac{F_{c}}{2500}$$

where *R_c_* is the compressive strength (MPa), *F_c_* is the load recorded at the point of fracture (N) and 2500 is the area (mm^2^) that the load was applied to, i.e., 50 × 50 mm. 

### 2.5. Scanning Electron Microscope (SEM) Imaging

Fragments from the compressive strength testing were collected and used for SEM imaging. Pieces no greater than 10 mm in any dimension were coated using carbon evaporation under vacuum. The device used was a Zeiss GeminiSEM Field Emission Scanning Electron Microscope, which allowed for multiple methods of imaging to be used. Images of the fracture surface were obtained using secondary electron (SE) imaging and electron backscatter diffraction (BSE).

SE imaging is the detection of low energy electrons emitted from the surface of the specimen exposed to the high energy electron beam and can be used to determine specimen topography. BSE is the detection of primary electrons that have been diffracted by an angle greater than 90°. The angle at which the electron has been diffracted is related to the atomic structure of the scattering material and can be used to reveal changes in specimen composition [30]. 

## 3. Results and Discussion

### 3.1. XRD Analysis

XRD diffractograms of the OPC and PCC-A blended cement samples tested were analysed using Rietveld Refinement, using Profex/BGMN [31] in accompaniment with structure data from ICSD [32]. Table 1 and Table 2 provide, in standard notation, the phases identified in the cement clinker and the oxides present in those phases. The PLC used for comparison interestingly had a calcium carbonate content of 18.27%, which was more than the maximum amount of PCC-A blended with OPC that was tested (15%). 

The chemical composition of the PCC-A used in the blended cements is summarised in Table 3. This PCC-A was comprised of calcite with a very small, residual salt content remaining from the production process. No other CaCO_3_ polymorphs were identified using Rietveld Refinement.

The NaCl content attributed to the PCC-A dropped to 0.02% when a 10% blended PCC-A cement was produced. This is the same as the NaCl content in the fresh tap water used to cure the test samples. Although the presence of NaCl can lead to an increased compressive strength, at this very low level it would be limited to no more than ~0.3%. A 10+% relative increase in the 28-day compressive strength of PCC-A blended cements, therefore, cannot be attributed to the presence of NaCl. The logical conclusion is that it is attributed to the use of PCC-A.

XRD analysis provides a powerful tool for identifying the purity of PCC-A samples and will be of great benefit in future when different PCC-A polymorphs and granularities are used.

### 3.2. Determination of Strength

After 7 days of curing, the PCC-A cement blends showed a reduction in strength with the carbon-negative Precipitated Calcium Carbonate (PCC) content above 4%. However, after 28 days, an increase in strength was observed up to 10% PCC. This increase in strength was accompanied by a decrease in the workability of the cement paste, tentatively attributable to the formation of carboaluminate phases, which act as a strong binder between the cement paste, unreacted PCC-A and, in the case of concrete, aggregates [13].

**Figure 3:**
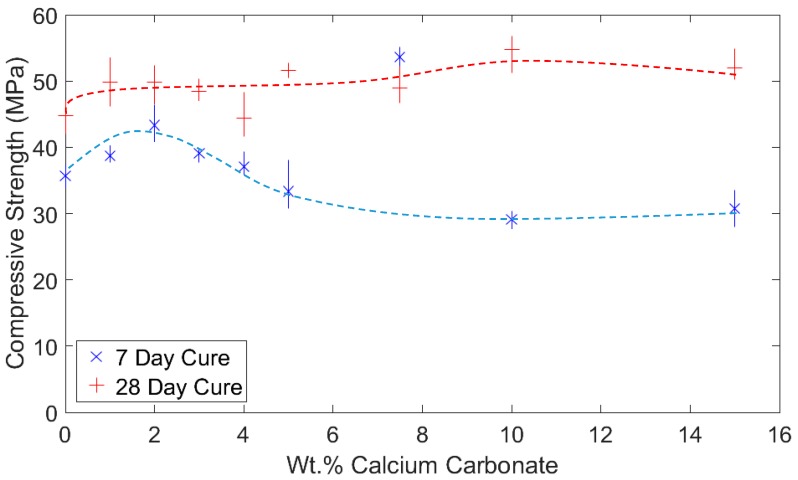
Compressive strength of cements containing PCC-A after 7 and 28 day of curing, with an increase in strength of up to 4% content in the former and 10% content in the latter, respectively.

The complete set of results from the compressive strength testing is presented in Figure 3. It can be seen that an increase in strength is gained after 7 days of curing for PCC-A content between 1 and 4%. Beyond 4% PCC-A, the strength of the cement starts to decrease as the PCC-A content increases. After 28 days, the strength increase resulting from the inclusion of PCC-A continues up to and beyond 15%, reaching a maximum strength of 54.8 MPa at 10% PCC-A content. This represents an increase of approximately 20% compared to the OPC reference. A PLC sample was separately tested and found to have a compressive strength of 18.1 MPa and 22.3 MPa at 7 and 28 days, respectively.

Since all the tests were carried out on cement pastes, the expected 52.5 MPa compressive strength of the OPC was not reached—this value is normally attributed to mortar strength.

Future work on strength will require testing samples at lower water/cement (w/c) ratios and increasing the curing time up to 365 days. OPC, PLC and 10% PCC-A will also be tested at a similar workability, as is shown in Section 3.3. The use of PCC-A in mortars will be studied, as the formation of carboaluminate phases has been documented to have strong bonding properties between aggregates and cement paste [13].

### 3.3. Rheological Analysis

**Figure 4:**
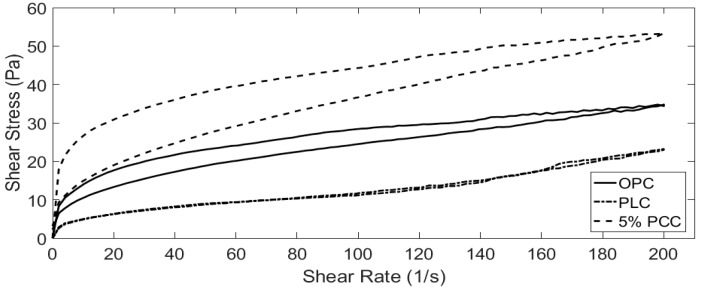
Shear stress measured in cement samples with water/cement (w/c) ratio = 0.5 as rate of shear increases.

**Figure 5:**
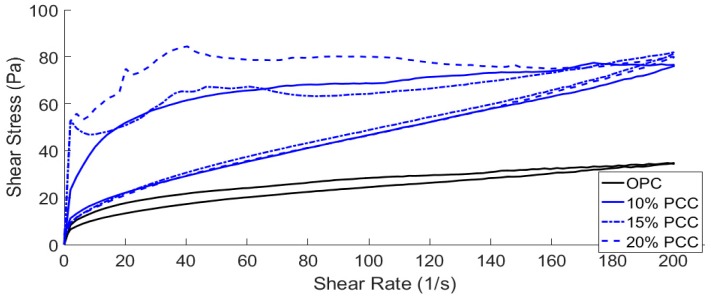
Shear stress measured in cement samples containing high PCC-A content with w/c = 0.5 as shear rate increases.

**Figure 6:**
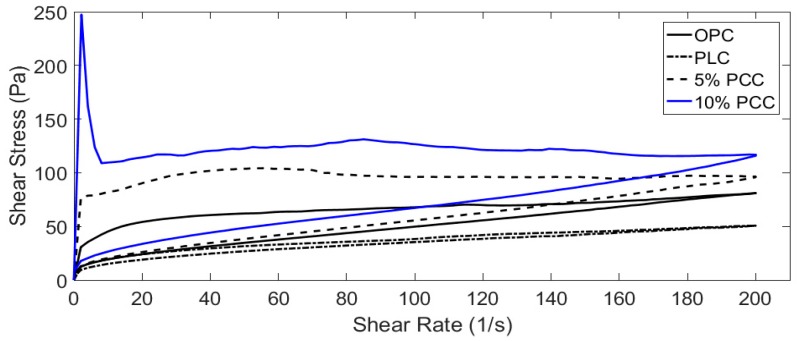
Shear stress in cement samples at w/c = 0.4. The 15 and 20% PCC-A samples became unworkable at this w/c without the use of plasticiser and have been omitted.

**Figure 7:**
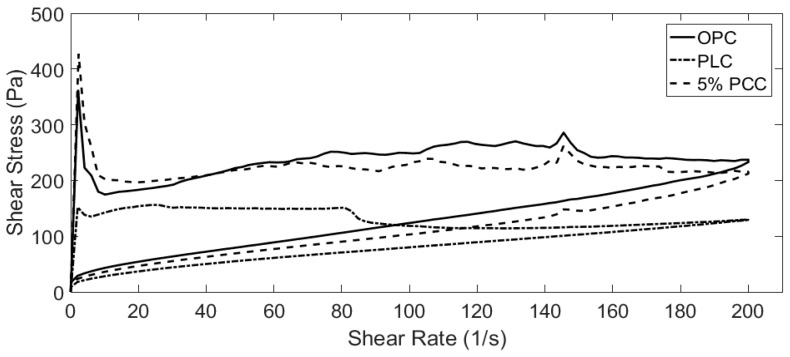
Shear stress in cement samples containing w/c = 0.3. The 10% PCC-A became unworkable at this w/c and are excluded.

Cement pastes with water/cement ratios of 0.5, 0.4 and 0.3 were tested using the method described in Section 2.3. Data were recorded using a Brookfield R/S+ rheometer in conjunction with the Rheo3000 software package. The addition of PCC-A to cement created a more viscous paste (Figure 4, Figure 5, Figure 6 and Figure 7), whereas the PLC tested for comparison was less viscous (Figure 4, Figure 6 and Figure 7).

As the w/c ratio of the cement pastes decreases, the PCC-A cement samples over 10% became too viscous for the rheometer to start and are not included in Figure 6 and Figure 7. This is likely due to the large shear stress that can be observed in the initial part of the tests, where setting of the cement paste starts early. However, the pastes then break down quickly and become fluid and workable again. The overt rise in viscosity is termed “false set” and can sometimes be overcome by using admixed plasticiser. The false-setting compositions, assuming that normal strength gain occurs at later stages, might actually be useful in developing sprayed concretes—exploring this is a task for the future. However, these compositions would not be useful in conventional construction. 

**Figure 8:**
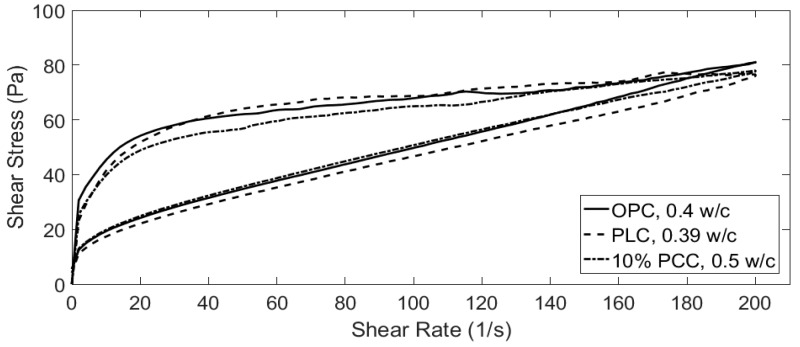
Workability of three different blends to determine the w/c ratio for which OPC, PLC and 10% PCC-A samples exhibit similar rheological properties.

As the 10% PCC-A cement samples showed the greatest compressive strength of those tested (see Figure 3), cement pastes of OPC and PLC that provided a similar workability by adjusting the w/c ratio were found. These w/c ratios are shown in Figure 8. The volume of water required for a 10% PCC-A to obtain a similar workability to OPC is 25% higher. For PLC, the required volume is 2.5% lower. Further work is clearly needed to support the wide use of PCC-A cement blends in future.

Further work is planned and will be carried out on the rheology of PCC-A cement blends to definitively establish why they behave in a manner dissimilar to what is known for PLC.

### 3.4. Scope for Emission Reduction of PCC-A Cement Blends

The reduction in CO_2_ emissions through the use of PCC-A can be calculated as follows:

CO_2_ reduction, C = w_opc_ · m_opc_ + w_pcc_ · m_pcc_, (2)

where w_opc_ and w_pcc_ are the mass fractions of OPC and PCC-A respectively, and m_opc_ and m_pcc_ is the mass, in grams, of the CO_2_ emitted in the production of 1 kg of the respective product. The final potential reduction in cement is then calculated using the reduction in clinker required to achieve the same compressive strength as OPC. The final reduction in CO_2_ emissions is then found as:

% reduction CO_2_ = (1 − (C_i_ · (R_c-opc_/R_c-i_))/C_opc_)) · 100, (3)

where C_i_ is the CO_2_ reduction of a PCC-A with i = 1–15 wt.% PCC-A, C_opc_ is the CO_2_ emitted from OPC production, R_c-opc_ is the mean compressive strength of OPC and R_c-i_ is the mean compressive strength of the PCC-A blended cement with i = 1–15%. For example, the reduction in CO_2_ for the 10% PCC-A cement is calculated as follows:

CO_2_ reduction, C_10%_ = 0.9 · 951.5 gCO_2_/kg + 0.1 · (−100) gCO_2_/kg, (4)

C_10%_ = 846.35 gCO_2_/kg, (5)

% reduction CO2 = (1 − (846.35 gCO_2_/kg · (44.8 MPa/54.8 MPa))/ 951.5 gCO_2_/kg)) · 100, (6)

% reduction CO2 = 27.3%.(7)

**Figure 9:**
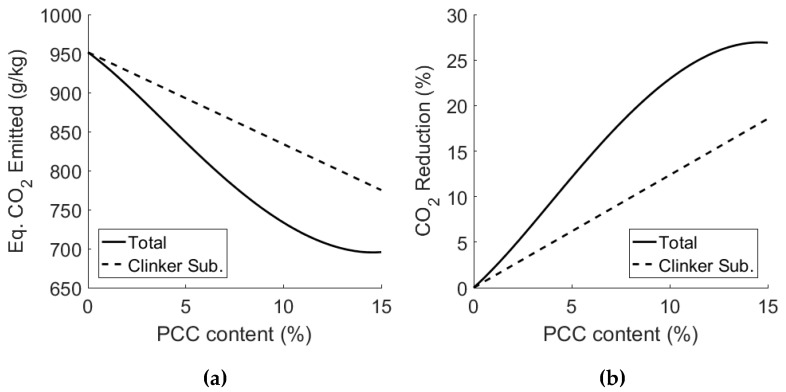
Clinker substitution with PCC-A produced using NaOH: (**a**) CO_2_ emissions of PCC-A cement blends with increasing PCC-A content and strength considerations, and (**b**) percentage reduction in CO_2_ emissions of PCC-A cement blends through clinker substitution and strength gain.

The reduction in CO_2_ emission attributed to the use of PCC-A as a carbon-negative clinker substitute, excluding any embodied CO_2_ reduction that may be attributed to decarbonising the cement manufacturing process (as suggested in Figure 1), is summarised in Figure 9. The values for clinker substitution alone are displayed as dashed lines; the total reduction values, incorporating the effect of the strength increase of PCC-A cement blends and the associated reduction in the structural member size/volume, are represented as solid lines.

Traditionally, the production of one kilogram of OPC clinker emits on average ~950 gCO_2_ [33]. The production of one kilogram of PCC-A sequesters ~100–350 gCO_2_, depending on the alkali used in the capture process. To date, the carbon mitigation of two alkalis has been examined—sodium hydroxide and, to a limited extent, ammonia. The production of sodium hydroxide has a carbon intensity (i.e., the weight ratio of CO_2_ emitted in production per unit weight of product made) of ~870 gCO_2_/kg [34] and ammonia has a carbon intensity of ~1,600 gCO_2_/kg [35]. However, there is a body of evidence to suggest that 80% of ammonia used in the reaction could be regenerated and reused, and so the carbon intensity could be reduced to 667 gCO_2_/kg due to its lower material demand during the CO_2_ capture process. For further information, see the Technology Centre Mongstad (TCM) Chilled Ammonia Process (CAP), which is based on the chemistry of the NH_3_-CO_2_- NH_3_ system [36].

**Figure 10:**
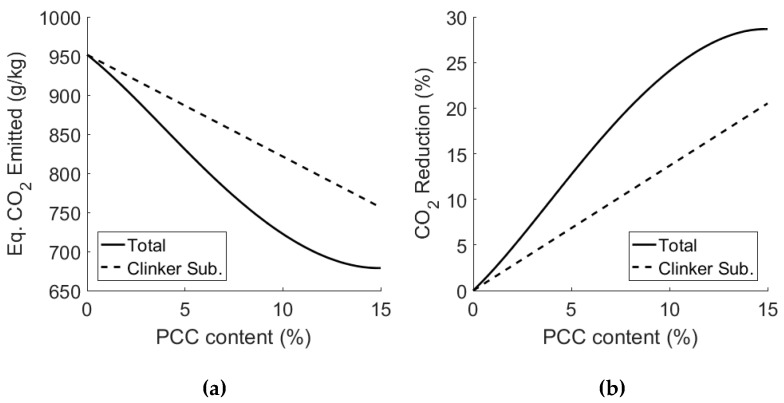
Clinker substitution with PCC-A produced using NH_3_: (**a**) CO_2_ emissions of PCC-A cement blends with increasing PCC-A content and strength considerations, and (**b**) percentage reduction in CO_2_ emissions of PCC-A cement blends through clinker substitution and increase in strength.

The final CO_2_ sequestered using either sodium hydroxide or ammonia is then 100 gCO_2_ or 350 gCO_2_, respectively. The PCC-A used in the test results that have been reported was produced using sodium hydroxide, and the values in Figure 9 reflect that. Figure 10 shows the potential reduction in emissions if recovered ammonia is used as the alkali in the PCC-A production process. Alkalis from waste could potentially offer equally low carbon intensity alternatives.

The introduction of PCC-A blended cements could have a significant impact on the carbon footprint of the cement industry (and with that the built environment), due to the reduction in CO_2_ emissions in the PCC-A production process and the subsequent substitution of clinker using PCC-A. As shown in Figure 9, the 15% PCC-A blended cement made using a NaOH CAPCON process provided a reduction in emissions of 27% compared to OPC. A PCC-A made using an ammonia process would deliver a 30% reduction. Future work on the use of PCC-A will involve a detailed from cradle-to-grave Life Cycle Assessment (LCA) of PCC-A cement blends, and it will study how they compare against other cements and cement blends in terms of overall performance, resistance to deterioration and cost.

### 3.5. SEM Imaging

**Figure 11:**
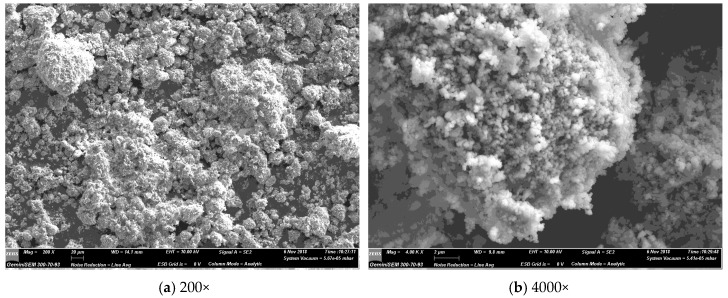
SEM micrographs of the PCC-A that was used in cement blends using secondary electron (SE) detection. Grain size was estimated to range from less than 100 nm to 20 µm. (**a**) 200× magnification of PCC-A and (**b**) 4000× of the same PCC-A sample.

**Figure 12:**
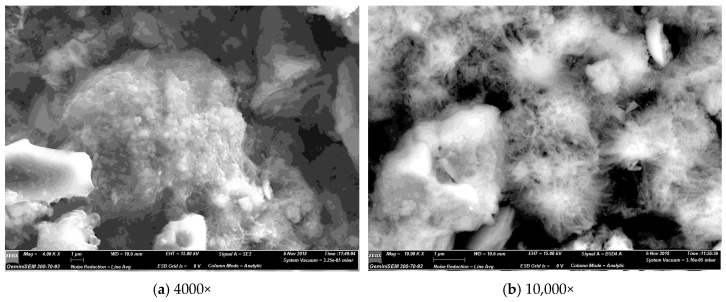
SE images showing fracture surface topography in a sample of the 15% PCC-A cement blend at different magnifications. (**a**) 4000× magnification and (**b**) 10,000× magnification.

**Figure 13:**
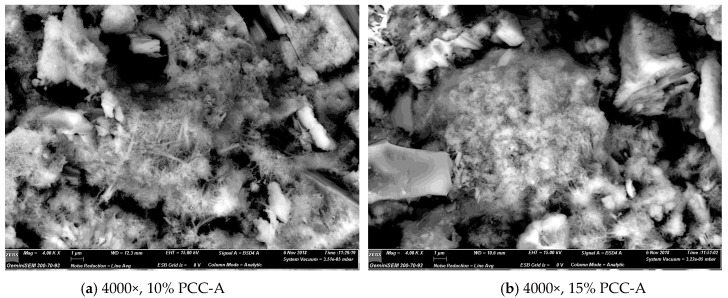
Electron backscatter diffraction (BSE) images displaying various phases on fracture surface. Each phase present is displayed by different intensities of black and white, with black representing unreacted PCC-A fragments. (**a**) 4000× magnification of 10% PCC-A cement blend and (**b**) 4000× magnification of 15% PCC-A cement.

SEM micrographs were obtained using a secondary electron detector. Figure 11 is of the non-optimised PCC-A that was used to produce the samples and test results reported in this paper, at progressively higher magnifications. Figure 12 presents and the backscatter electron detector images of the fracture surface of the 15% PCC-A cement blend. Both large and small PCC-A crystals were identified on the fracture surface of the sample using SEM imaging, the latter possibly due to the increased surface area for reaction required for the formation of carboaluminate phases. Figure 13 is a backscatter detector image of the fracture surface of the 10% and 15% PCC-A cement blends. 

SEM imaging is a useful tool in determining the presence of PCC-A in cement blends and for grain size determination. Further work to determine the presence of carboaluminate phases and the progression of phase composition is planned for samples cured from 1, 7, 28 and 365 days.

## 4. Conclusions

PCC-A, made using flue gas CO_2_ in CCM’s CAPCON process, used as a Portland cement admixture, leads to an increased compressive strength that peaks at 10 wt.% PCC-A content. Combined with the carbon-negative nature of PCC-A, the resulting reduction in the clinker factor and the cumulative carbon content, the carbon footprint of a PCC-A blended cement containing 15% PCC-A is equivalent to ~73% of that of the OPC average.

The strengths of PCC-A cement blends increase with PCC-A content, but the workability reduces, and, speculation notwithstanding, a full and detailed understanding of the processes that are involved is needed.

To achieve the same level of workability as an OPC paste, a PCC-A cement paste requires 25% more water, which is not how a PLC cement behaves (requiring 2–5% less water).

Future work to be undertaken on the effects of PCC-A in blended cements will include:

- The testing of PCC-A for use in mortars to determine the binding effects on aggregates;
- Examination of PCC-A cements after long curing times to study the evolution of carboaluminate phases;
- Investigation of the inverse effects on rheology compared with PLC;
- Full LCA of PCC-A blended cements using more alkali-efficient mineralisation processes;
- Preparation of PCC-A blended cements containing polymorphs of PCC-A to determine if crystal structure plays a role in reactivity during hydration.

## Figures and Tables

**Table 1:** Quantitative analysis of Ordinary Portland Cement (OPC) used in tests and oxides present.

Mineralogical Composition	Phase Wt. %	Chemical Composition	Oxide Wt. %
C_3_S	59.65	SiO_2_	20.28
C_2_S	15.24	Al_2_O_3_	4.71
C_3_A	11.81	Fe_2_O_3_	3.27
C_4_AF	8.65	CaO	67.13
C$\bar{S}$H_2_	4.65	SO_3_	2.54
		MgO	0.67
		K_2_O	1.40

**Table 2:** Quantitative analysis of Portland limestone cement (PLC) and oxides present.

Mineralogical Composition	Phase Wt. %	Chemical Composition	Oxide Wt. %
C_3_S	60.11	SiO_2_	15.23
C_2_S	7.66	Al_2_O_3_	3.33
C_3_A	9.42	Fe_2_O_3_	2.75
C_4_AF	1.03	CaO	64.40
C$\bar{S}$H_2_	3.51	SO_3_	1.41
CaCO_3_	18.27	MgO	0.64
		K_2_O	0.83
		CO_2_	11.41

**Table 3:** Quantitative analysis of PCC-A used in cement blends.

Compound	Wt. %
Calcite	99.8
Aragonite	0.0
Vaterite	0.0
Halite, NaCl	0.2
